# Directed Self-Assembly of Diamond Networks in Triblock Terpolymer Films on Patterned Substrates

**DOI:** 10.1021/acsami.3c10619

**Published:** 2023-11-21

**Authors:** Doha Abdelrahman, René Iseli, Michimasa Musya, Butsurin Jinnai, Shunsuke Fukami, Takeshi Yuasa, Hiroaki Sai, Ulrich B. Wiesner, Matthias Saba, Bodo D. Wilts, Ullrich Steiner, Justin Llandro, Ilja Gunkel

**Affiliations:** † Adolphe Merkle Institute, University of Fribourg, Chemin des Verdiers 4, 1700 Fribourg, Switzerland; ‡ Laboratory for Nanoelectronics and Spintronics, Research Institute of Electrical Communication, Tohoku University, 2-1-1 Katahira, Aoba-ku, Sendai 980-8577, Japan; ¶ WPI Advanced Institute for Materials Research, Tohoku University, 2-1-1 Katahira, Aoba-ku, Sendai 980-8577, Japan; § Center for Science and Innovation in Spintronics, Tohoku University, 2-1-1 Katahira, Aoba-ku, Sendai 980-8577, Japan; ∥ Center for Innovative Integrated Electronic Systems, Tohoku University, 468-1 Aramaki Aza Aoba, Aoba-ku, Sendai 980-0845, Japan; ⊥ Inamori Research Institute for Science, Kyoto 600-8411, Japan; # Department of Materials Science and Engineering, Cornell University, 214 Bard Hall, Ithaca, New York 14853-1501, United States; @ Department of Chemistry and Physics of Materials, University of Salzburg, Jakob-Haringer-Str. 2a, Salzburg 5020, Austria; △ Swiss National Center of Competence in Research (NCCR) Bio-Inspired Materials, University of Fribourg, Chemin des Verdiers 4, 1700 Fribourg, Switzerland

**Keywords:** block copolymer self-assembly, alternating diamond, chemical patterning, solvent vapor annealing, templated fabrication, nanostructured single diamond gold networks

## Abstract

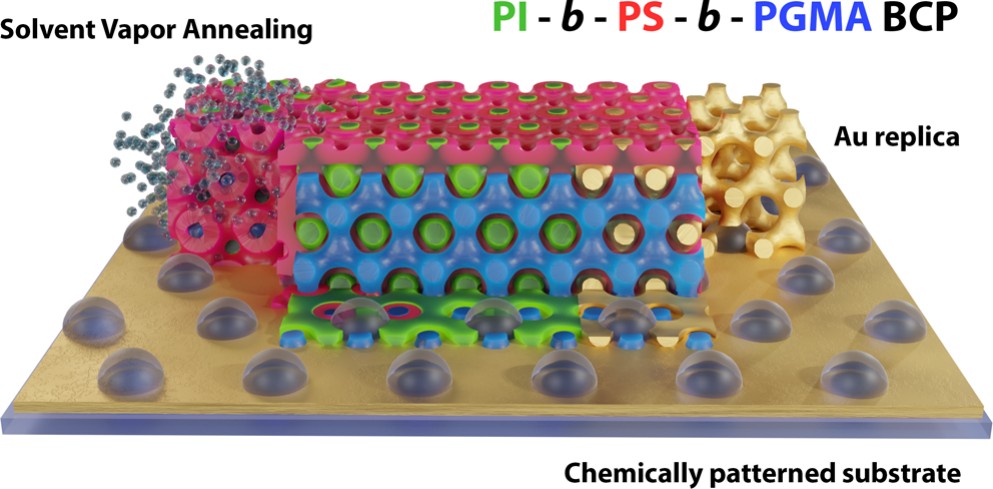

Block copolymers (BCPs) are particularly effective in creating soft nanostructured templates for transferring complex 3D network structures into inorganic materials that are difficult to fabricate by other methods. However, achieving control of the local ordering within these 3D networks over large areas remains a significant obstacle to advancing material properties. Here, we address this challenge by directing the self-assembly of a 3D alternating diamond morphology by solvent vapor annealing of a triblock terpolymer film on a chemically patterned substrate. The hexagonal substrate patterns were designed to match a (111) plane of the diamond lattice. Commensurability between the sparse substrate pattern and the BCP lattice produced a uniformly ordered diamond network within the polymer film, as confirmed by a combination of atomic force microscopy and cross-sectional imaging using focused ion beam scanning electron microscopy. The successful replication of the complex and well-ordered 3D network structure in gold promises to advance optical metamaterials and has potential applications in nanophotonics.

## 1 Introduction

Block copolymer (BCP) self-assembly is a well-established method for efficiently producing nanostructured morphologies such as spheres, lamellae, cylinders, and 3D network morphologies such as the gyroid.^1^ This morphological versatility, combined with widely tunable dimensions, has allowed the tailoring of material properties in various applications, including nanolithography,^2^ optical metamaterials,^3,4^ and nanophotonic materials.^5,6^ In films,^7^ BCP self-assembly creates surface patterns that can be used directly to guide the assembly of other molecules,^8^ such as proteins.^9^ However, many BCP-based functional materials are made by transferring the self-assembled nanostructures created in a BCP film after selectively removing one of the blocks.^10^ The resulting nanoporous film can be used either as a mask to pattern the underlying substrate or as a template for the deposition of inorganic materials.^4,11,12^

The functionality of the resulting materials is closely related to the structure of the BCP template, particularly its degree of order and the presence of defects. For example, the linear dichroism observed in large grains of gyroid-structured metamaterials made from BCP templates^13^ disappears in samples with small grain sizes.^14^ However, structural defects limit the grains in BCP films, i.e., the regions of uniform translational and rotational order.^15^ Therefore, it is essential to heal defects and increase grain size before BCP films are used as templates.

An efficient method for grain coarsening in BCP films is solvent vapor annealing (SVA), which introduces solvent vapor to plasticize the BCP film,^16^ thereby significantly increasing the mobility of the BCP chains and thus allowing structural defects to heal.^17^ However, to remove defects altogether, the BCP self-assembly must be directed by an external field.

Annealing BCP films in external electric^18^ or magnetic fields^19^ or under shear^20−23^ macroscopically orients the grains, creating long-range order with local defects remaining at significantly reduced density. Annealing BCP films on lithographically defined substrate patterns can produce defect-free structures.^24^ The directed self-assembly (DSA) of BCP films on topographically patterned substrates aligns the grains with respect to the topographic features, thereby removing defective grain boundaries.^25^ Using substrates with lithographically defined chemical patterns ensures precise registration of the chemically heterogeneous BCP structure on the substrate, replicating the underlying pattern and eliminating defects.^26^ Chemical and topographical patterning of substrates has enabled the fabrication of linear and highly intricate single-layer films over large areas by using spherical, lamellar, and cylindrical BCP films. The size of the substrate pattern is the primary limitation to achieving single-grain BCP films, while using sparse patterns helps to reduce lithographic requirements. However, despite advances in patterning 3D structures, the focus has been primarily on 1D and 2D multilayer morphologies, leaving the behavior of continuous 3D BCP networks on patterned substrates largely unexplored.^24^

The interest in 3D BCP networks has rapidly grown as well-ordered 3D bicontinuous structures provide materials that exhibit a wide range of exceptional optical and magnetic properties, including a tunable effective optical behavior,^14,27^ a strong chiro-optical response,^28^ and multiple equivalent magnetization configurations.^29^ For these applications, controlling the self-assembly of 3D bicontinuous cubic BCP morphologies is crucial to generate macroscopic arrays of these 3D nanostructures. DSA of a double gyroid was previously demonstrated in a sub-100 nm thick diblock copolymer film on a topographical grating-patterned substrate, resulting in large double gyroid grains (>10 μm^2^).^30^ While this is the only report of a bicontinuous network-like morphology, other 3D structures with large grain sizes have been fabricated by DSA, such as sphere packings.^31^

Interpenetrating network morphologies have practical advantages, for example, in photonic crystal bandgap^32^ and optical and magnetic metamaterial engineering.^33,34^ Depending on the copolymer used, these are either *alternating* dual networks, where the two networks consist of different polymers, or *double* networks, where both networks are made of the same polymer. An overview of the most well-known alternating and double polycontinuous geometries and their symmetries are listed in Table 1 of ref (35). Alternating and double gyroid structures are extensively studied equilibrium morphologies in triblock terpolymer and diblock copolymer bulk systems, respectively, both in bulk systems^36^ and films.^37−40^ In contrast, alternating diamond morphologies are predicted to occur only in a small phase-space region near the order–disorder transition in triblock terpolymers.^41,42^ Recently, the an alternating diamond (*Fd*3̅*m*, space group no. 227)^43^ has been generated in a triblock terpolymer by SVA.^44^ This particular system is employed in the present study.

Here, we report the surface-induced control of a BCP diamond lattice during solvent vapor annealing, both in-plane and in the normal direction of the structure-inducing surface. The experiments encompass three essential elements: (1) BCP self-assembly into a diamond lattice even under unfavorable external constraints such as physical confinement and substrate interactions; (2) the supply of a substrate pattern with suitable wettability contrast with respect to the polymer blocks; and (3) an annealing protocol that enables the selective nucleation of BCP self-assembly at the guiding substrate while providing sufficient polymer mobility for the surface-induced structure to propagate away from the substrate.

## 2 Results

**Figure 1:**
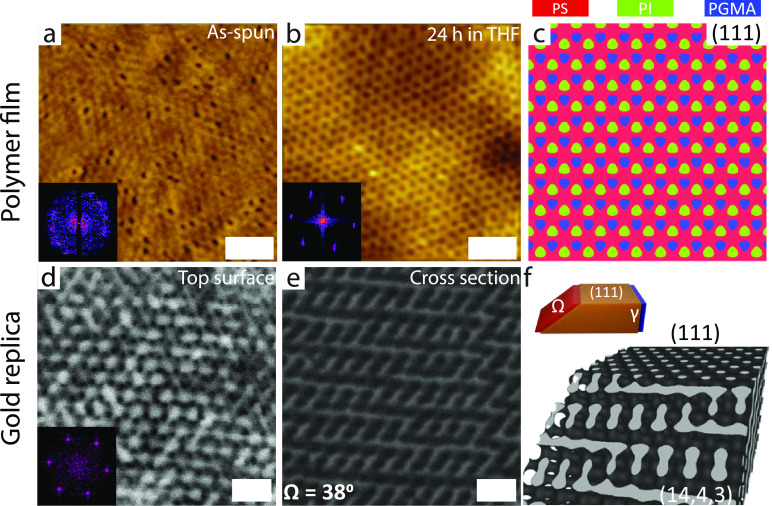
Atomic force microscopy (AFM) images of ca. 600 nm thick ISG films (a) directly after spin coating and (b) after swelling in THF to a maximum swelling ratio of 2.2 and subsequent slow drying for 24 h. (c) A (111) plane of the level-set diamond according to eq 1 with fill fractions *f*_PI_ = 0.29 (green), *f*_PS_ = 0.52 (red), and *f*_PGMA_ = 0.19 (blue). (d) SEM top-view and (e) FIB-SEM cross-sectional view of a gold replica of a 24 h solvent-annealed 600 nm thick ISG film. The cross-section was taken at an angle Ω = 38° relative to the top surface of the sample. (f) (111) and (14, 4, 3) cuts across a single diamond level-set model with a 30% fill fraction; the angle between the two planes is 35°. The insets in parts a, b, and d show the corresponding FFTs. The inset in (f) shows a 3D model for the FIB-SEM cuts with the different angles Ω and γ. Scale bars: (a,b) 200 nm and (c,e) 100 nm.

Our study uses a polyisoprene-*block*-polystyrene-*block*-poly(glycidyl methacrylate) (PI-*b*-PS-*b*-PGMA, ISG) triblock terpolymer, which was synthesized by sequential anionic polymerization as described previously.^44^ The ISG terpolymer has a molar mass of 67.4 kg mol^–1^ and volume fractions of *f*_PI_ = 0.29, *f*_PS_ = 0.52, and *f*_PGMA_ = 0.19. We have recently shown that ISG terpolymer films form a diamond-like morphology when exposed to tetrahydrofuran (THF) vapor, swelling up to a maximum swelling ratio of 2.1, followed by 44 h of drying.^44^ In this study, we used a similar SVA protocol but with a reduced drying time of 24 h for practical reasons. This adjustment was supported by the fact that the films used in this study were either thinner or had a thickness similar to those in our previous study on unpatterned substrates. The top surface of the ISG films was characterized by atomic force microscopy (AFM), where the poor ordering for the as-spun ISG film (Figure 1a) was significantly improved by SVA, as evidenced by a well-ordered array of hexagonal dots for the solvent-annealed ISG film (Figure 1b). The hexagonal pore arrangement is consistent with a (111) surface of the alternating diamond, where a crystal termination can be chosen such that the dark dots in the AFM images signify one of the minority phases (PI or PGMA), surrounded by the continuous PS phase (Figure 1c).

To allow for characterization by scanning electron microscopy (SEM), the structure formed by the PI phase was replicated in gold. This was achieved by selectively removing the PI phase from the dried ISG films and filling the voided structure with gold^45^ (see Experimental Section for details). The top-view and cross-sectional SEM images of the gold replica were compared to planes in a single diamond network described by the level-set equation (details in Experimental Section)
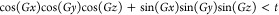
1where *t* ≈ 5/3(1/2 – *f*) depends on the fill fraction *f* of the network (*f* ≈ 0.3 in the present samples) and *G* = 2π/*a* is the reciprocal value of the lattice constant *a*. The top-view SEM image in Figure 1d shows a hexagonal arrangement of dots similar to the AFM observations (Figure 1b). Direct measurements and the fast Fourier transform (FFT) images in the insets confirm the real-space symmetry with a nearest neighbor distance (NND) between the dots of (40 ± 2) nm.

Cross-sectional imaging of the replicated gold network was performed by using focused ion beam scanning electron microscopy (FIB-SEM). Figure 1e displays an image of a section cut into the sample at an angle of Ω = 38° with respect to the film surface (illustrated in the inset of Figure 1f), which shows a distinct pattern of horizontal and diagonal struts. Despite some noticeable distortion, this strut pattern agrees well with the (14, 4, 3) plane in the single diamond level-set model (Figure 1f), which exhibits a 35° angle relative to the (111) diamond of the top surface.

The FFT measures of the nearest neighbor distance in the solvent-annealed ISG terpolymer film and its gold replica shown in Figure 1 enabled the design of patterned substrates for the DSA of the ISG triblock terpolymer. While it is possible to write a point pattern with a 40 nm NND using electron-beam lithography, large-area patterning with this resolution is time-consuming and not robust. Instead, we e-beam fabricated sparse hexagonal substrate patterns with 2 to 3 times the NND of the hexagonal terpolymer patterns in Figure 1b. As previously demonstrated, sparse substrate patterns allow the DSA of hexagonal BCP arrays when their periodicity is commensurate with the BCP pattern.^46^ The manufactured patterned substrates consist of Au-covered silicon wafers onto which ca. 20 nm thick silica patches were deposited by electron-beam writing of a silsesquioxane-containing resist (for details, see the Experimental Section). Note that the relatively shallow topography of the substrate, characterized by a silica patch height approximately 2 to 3 times smaller than the NND within the diamond structure, may still affect the formation of the diamond grains.^47,48^

Water contact angle measurements on homogeneously deposited films yielded values of ∼25° for SiO_2_ (∼25 nm thick SiO_2_ film on Si) and ∼62° for Au (∼20 nm thick Au film on Si). Note that while Au presents a surface with very high surface energy in vacuum, Au surfaces are readily contaminated by the adsorption of hydrocarbons under ambient conditions, and contact angles larger than 40° are routinely measured for water in contact with gold surfaces.^49^ For the present study, this results in a more polar point pattern surrounded by a less polar background, in agreement with the choice of patterned substrate motivated above.

**Figure 2:**
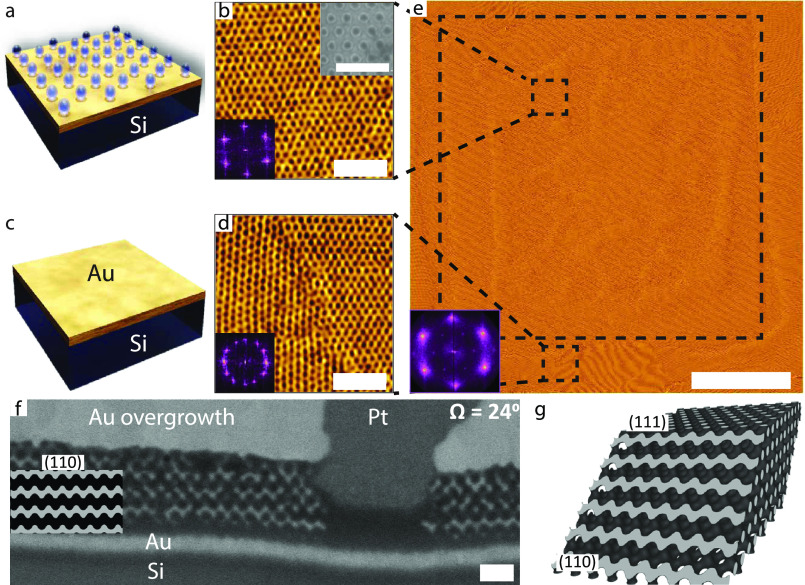
Illustration of (a) the hexagonal silica dot pattern on top of a Au-coated Si wafer and (c) bare Au-coated Si outside the patterned region. High-magnification AFM images of a solvent-annealed 120 nm thick ISG film on top of (b) a silica pattern ((71 ± 3) nm NND, (50 ± 2) nm diameter; see SEM image in the upper-right inset), and (d) a homogeneous Au-coated Si. The corresponding low-magnification AFM image in (e) shows a single uniform Moiré texture inside the area with the hexagonal pattern of silica patches marked with a large dashed square and multiple Moiré patterns of different orientations outside the silica pattern. The two smaller dashed squares mark the areas where the high-resolution images in (b) and (d) were taken. The insets in (b), (d), and (e) show the corresponding FFTs. The FFT in (e) was taken from a high-resolution AFM image of the ISG film atop the patterned region (Figure S1). (f) Cross-sectional SEM image of a gold replica of a 120 nm thick solvent-annealed ISG film sliced at an angle of Ω = 24° with respect to the sample surface. The inset shows a (110) cut through a level-set single diamond with a fill fraction of 30%. (g) 3D view of the corresponding level-set diamond model sliced through (111) and (110) planes; the angle between these planes is 35°. Scale bars: (b,d), upper-right inset in (b) 300 nm, (e) 5 μm, (f) 100 nm.

Figure 2a–e shows the surface structure of a 120 nm thick ISG film that was annealed in THF vapor (maximum swelling ratio 2.2) and then dried for 24 h. The substrate consists of a lithographically patterned 16 × 16 μm^2^ region containing a hexagonal array of ∼20 nm thick silica patches with diameters of (50 ± 2) nm The NND of the silica patches is ∼(71 ± 3) nm, about twice the NND of the hexagonal polymer pattern above, providing commensurability between the two hexagonal patterns (Figure 2b). The dashed square in Figure 2e outlines the patterned region of the substrate. The single uniform Moiré pattern of the film structure indicates the absence of any lateral grain boundaries for the ISG on top of the patterned substrate region. However, outside of the dashed square on the unpatterned Au film, multiple Moiré patterns with different orientations indicate a polygrain structure with grain boundaries, as shown in the high-magnification AFM image in Figure 2d (see also Figure S1). The 24° cross-section of the Au replica of a 120 nm thick solvent-annealed ISG film (Figure 2f) aligns closely with the (110) plane in the single diamond level-set model (Figure 2g). However, this plane is oriented at an angle of 35° relative to the (111) diamond top surface, suggesting some angular distortion in the network.

**Figure 3:**
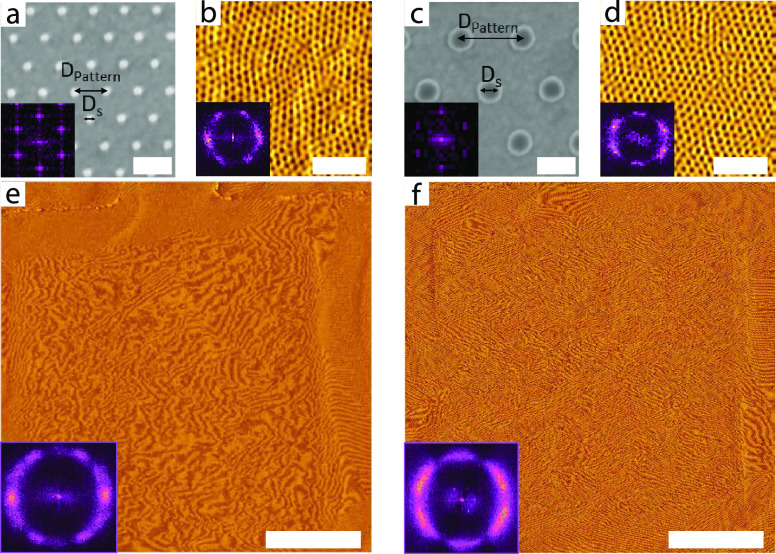
AFM images of a 120 nm thick solvent-annealed ISG film with identical conditions to Figure 2 but different hexagonal silica patterns. (a,c) SEM images of the substrate patterns with (71 ± 3) nm NND, (25 ± 1) nm diameter and (134 ± 3) nm NND, (60 ± 2) nm diameter, respectively. (b,d) High-resolution AFM images of the solvent-annealed ISG films atop the patterns shown in (a,c) show distortions in the hexagonal lattice that give rise to the distorted Moiré patterns in (e,f), respectively. The insets in parts (a–d) show the corresponding FFTs, while the insets in parts (e,f) show the FFT of high-resolution AFM images of the patterned areas (not shown). Scale bars: (a,c) 100 nm, (b,d) 300 nm, and (e,f) 5 μm.

While Figure 2 demonstrates the successful DSA of the ISG terpolymer film, it strongly depends on the substrate pattern. Figure 3 shows AFM images of the same ISG film as in Figure 2 on a different substrate location onto which different silica patterns were written. The substrate pattern in Figure 3a,b,e consists of a hexagonal array of 20 nm thick silicon patches with the same NND as in Figure 2 ((71 ± 3) nm) but with one-half of the patch diameters ((25 ± 1) nm). The substrate in Figure 3c,d,f has slightly larger diameter silica patches than in Figure 2 ((60 ± 2) nm) and ca. 3 times the NND ((134 ± 3) nm) of the ISG terpolymer pattern. The Moiré patterns on the patterned substrate regions in Figure 3e,f are much less regular than that in Figure 2e, exhibiting multiple different orientations, which indicates an isotropic polygrain structure with many grain boundaries and defects.

Visual inspection of the high-magnification AFM images in Figure 3b,d and the FFTs in the insets reveals a hexagonal-like packing in the surface pattern of the ISG terpolymer film with similar NNDs (of (40 ± 2) nm) as in Figure 2. However, the surface patterns are distorted and exhibit several defects, especially along the observed grain boundaries. Not only do these ISG films lack the uniform translational and orientational order observed on the patterned silica in Figure 2e, but their Moiré patterns in Figure 3e,f also lack a similarly obvious grain pattern seen for the ISG films on the unpatterned Au surfaces in Figure 2e, corroborating in-plane distortions in the surface patterns of these ISG films.

**Figure 4:**
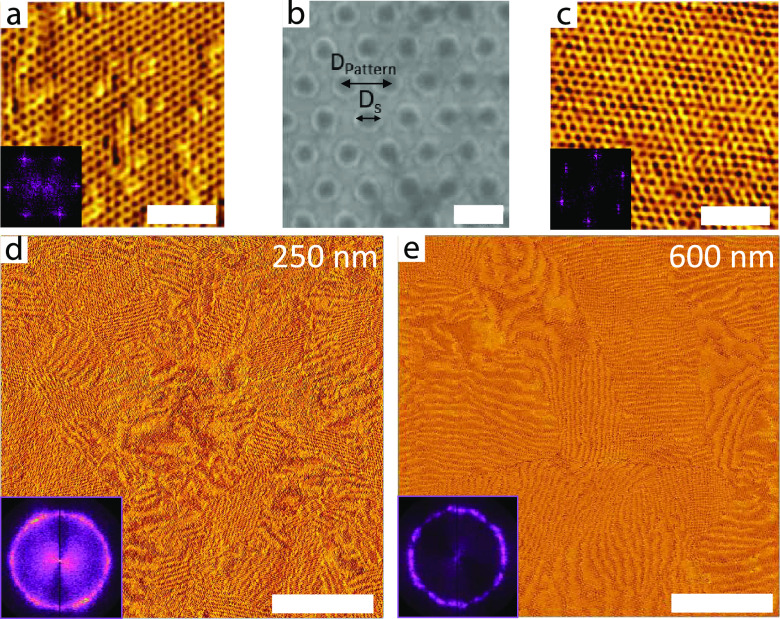
High-magnification AFM images of (a) 250 and (c) 600 nm thick solvent-annealed ISG films on Au substrates with the same silica pattern (shown in (b)) as in Figure 2 and the corresponding Moiré images in (d) and (e), respectively. While the high-magnification images and the corresponding insets confirm that local areas show well-ordered periodic morphologies, the Moiré images show an essentially isotropic polygrain structure, also visible in the FFTs of large-area high-resolution AFM images. Scale bars: (a,c) 300 nm, (b) 100 nm, (d,e) 5 μm.

In further experiments, the propagation of the ISG terpolymer’s DSA away from the patterned substrate was studied in thicker films. Figure 4 shows 250 and 600 nm thick films on the same silica-on-gold surface pattern as the film in Figure 2. In contrast to the thinner film, these thicker ISG films exhibit an in-plane polygrain structure that does not register with the underlying substrate pattern. In contrast to Figure 3e,f, the multiple differently oriented Moiré patterns in Figure 4d,e show an essentially isotropic grain structure similar to that of the film on an unpatterned surface. In particular, the lateral extent of the underlying patterned substrate areas is not visible in these images, indicating the absence of the patterned-substrate-induced DSA throughout the film’s thickness.

**Figure 5:**
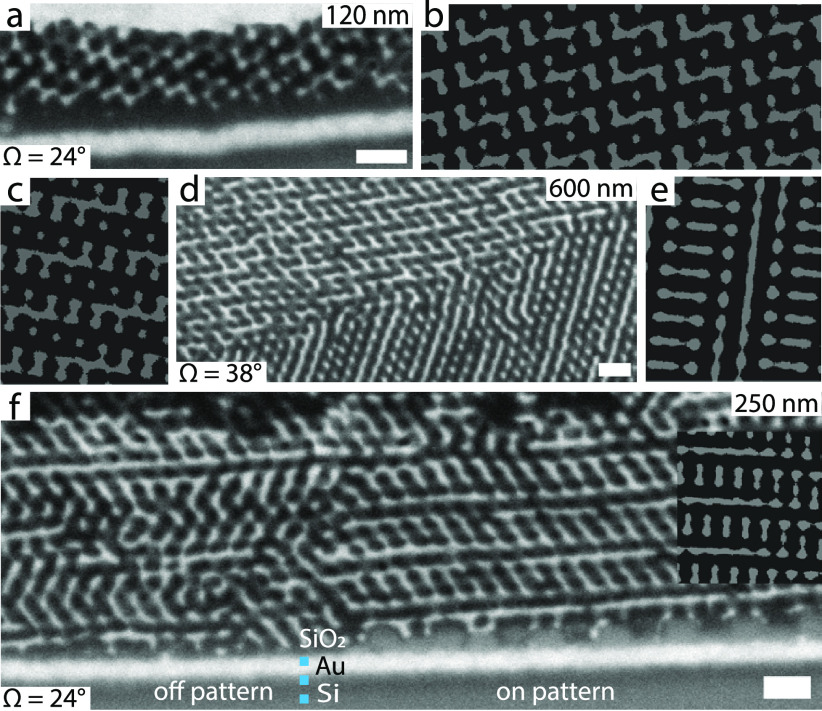
FIB-SEM images of cuts taken at different angles Ω relative to the sample surface through a Au replica of (a) 120, (d) 600, and (f) 250 nm thick ISG films. The diamond level sets (eq 1; fill fraction *f* = 0.375) show (b) (15, 7, 4), (c) (9, 2, 3), and (e) (10, 1, 1̅) planes, while the inset in (f) shows an (18, 3, 4) plane. The SEM image of the 250 nm thick ISG film (Ω = 24°) shows the transition from the unpatterned region of the Au-coated Si substrate to the area patterned with SiO_2_. The unstructured material on top of the film in (a) is an electrochemical overgrowth of Au. Scale bars: 100 nm.

Cross-sectional FIB-SEM imaging elucidates the lack of DSA in the thick films. As with the SEM images in Figure 1, the electron density contrast was enhanced by etching away the PI phase and backfilling it with Au. Figures 5 and S2 show SEM images of the Au replica for three ISG films with thicknesses of 120, 250, and 600 nm. The images of the top surfaces (Figure S2) are very similar to that in Figure 1d, showing predominantly hexagonal arrangements consistent with the characteristic (111) plane of the single diamond. For a 3D network such as the diamond, slicing the film at different angles with respect to the top surface creates many planes with distinct patterns, even if the network has a fixed out-of-plane orientation inside the film. Indeed, the images of the sections cut at different angles Ω (Figure 5a,d,f) and γ (Figure S3a,c,e) relative to the sample surface exhibit various distinct strut patterns. Each experimental pattern was matched with a plane of the diamond level-set model (eq 1, fill fraction^1^*f* = 0.375) as shown in Figure S3b,c,e,f (inset) and Figure S3b,d,f, respectively. While the topology of the modeled patterns resembles the SEM cross sections, the experimental patterns show noticeable distortions of the cubic diamond, both in the global pattern orientation with respect to the (111) substrate normal and the relative strut orientation. Additionally, the angles Ω and γ between the experimental cuts and the (111) top surface of the films differ from the corresponding angles in the level-set model (Table 1). This is generally consistent with a strong affine transformation of the (111) (normal compression combined with in-plane shear) caused by the drying of the film.^44^ Only the imaged region of the 250 nm film is not commensurable with a (111) orientation of the sample, as γ, Ω (Table 1), and the in-plane orientation of the matched cross sections (Figure 5f and S3d) show extreme deviations between the model and the experimental findings. In addition to the observed distortion, increasing the sample thickness led to a stronger tendency of the networks to form horizontal (i.e., parallel to the substrate) grain boundaries (Figure 5d), which were frequently observed for 600 nm samples and occasionally in the 250 nm samples but were absent in the 120 nm films. It is particularly interesting to directly observe the Au network on the silica patches and compare it with the corresponding network structure on the unpatterned Au surface, as shown in Figure 5f. Notably, two Au struts seem to be predominantly connected vertically to the silica patches, forming a well-ordered strut network that extends over several layers before reaching a grain boundary. In contrast, the network above the unpatterned regions lacks a similarly directed assembly.

**Table 1:** Angular Distortions Observed in Experimental Cross-Sectional Images of Films with Different Thicknesses Compared to (*hkl*) Planes in the Diamond Level-Set Model^a^

		*∠*(111)		
Thickness	Plane	Exp.	Sim.	Figure
120 nm	(15, 7, 4)	Ω = 24°	28°	5b
	(5, 4, 8̅)	γ = 90°	87°	S3b
250 nm	(18, 3, 4)	Ω = 24°	39°	5f
	(9, 2̅, 0)	γ = 90°	64°	S3d
600 nm	(9, 2, 3)	Ω = 38°	34°	5c
	(10, 1, 1̅)	Ω = 38°	55°	5e
	(1, 1, 2̅)	γ = 90°	90°	S3e

a *∠*(111) denotes the angle between the plane specified by the given (*hkl*) values and the (111) plane, while Ω and γ denote the angles of the experimental section with respect to the film surface as defined in Figure 1f.

## 3 Discussion

**Figure 6:**
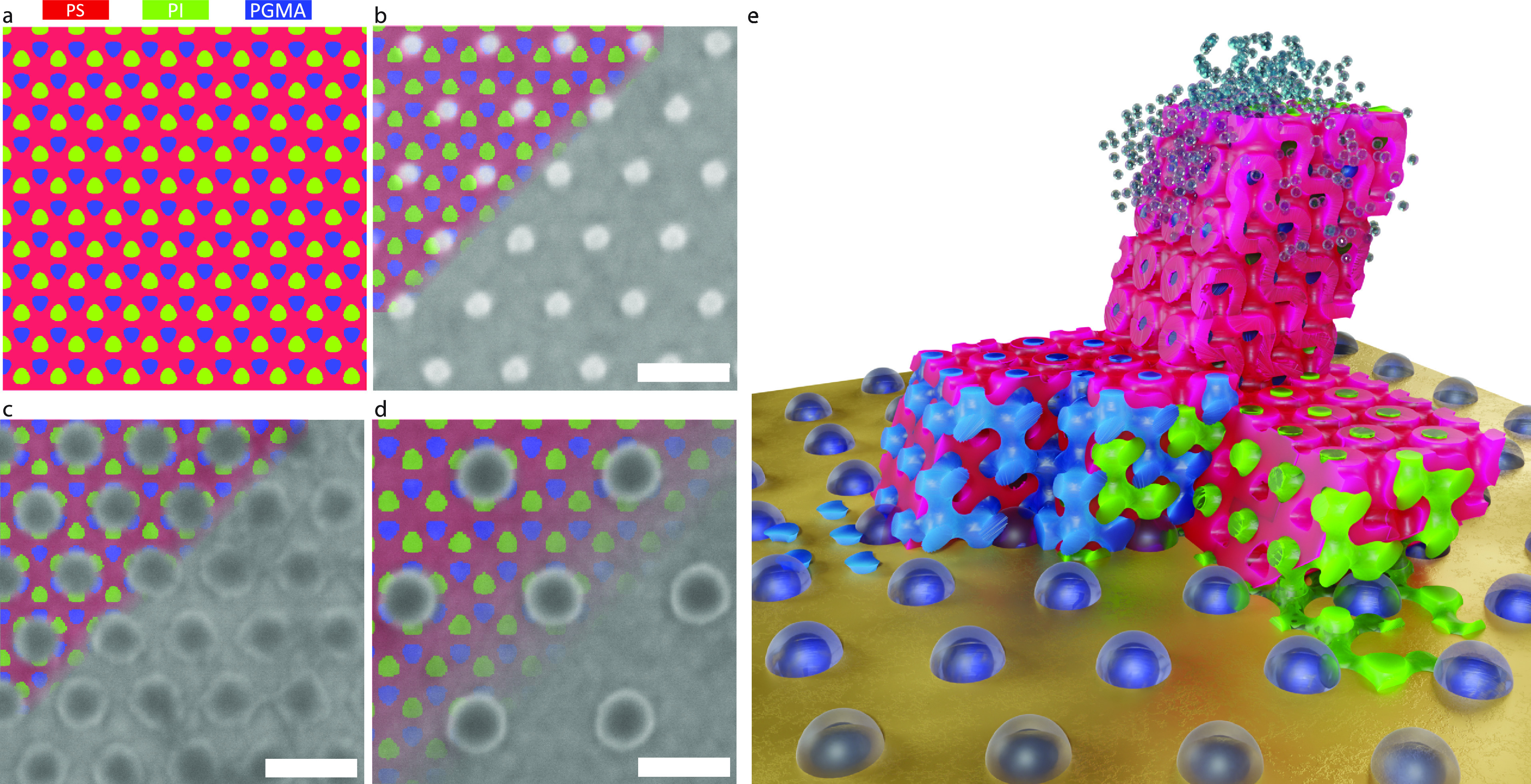
(a) (111) plane of the alternating diamond level-set model, eq 1, with fill fractions *f*_PI_ = 0.29 (green), *f*_PS_ = 0.52 (red), and *f*_PGMA_ = 0.19 (blue). (b–d) SEM images of the substrate patterns superposed with the (111) level set shown in (a). The dimensions of the substrate patterns are (b) (71 ± 3) nm NND, (25 ± 2) nm patch diameter, (c) (71 ± 3) nm NND, (50 ± 2) nm patch diameter, and (d) (134 ± 3) nm NND, (60 ± 2) nm patch diameter. The superposed level-set images are compressed by 13% in (b,c) and stretched by 12% in (d). Scale bars: 100 nm. (e) 3D schematic of the scenario in (c). The alternating diamond is cut to visualize the alignment of its two networks (blue and green) with the underlying silica pattern and the wetting of the struts on the silica patches (the red matrix is suppressed in the foreground for clarity). A smaller solvated layer is shown on top of the dry alternating diamond layer near the substrate.

The ISG triblock terpolymer in this study self-assembles into an alternating diamond morphology in films of various thicknesses. Using a fixed SVA protocol with a constant swelling ratio and drying times, cross-sectional SEM imaging revealed diamond networks on both patterned and unpatterned substrates (Figures 1, 2, and 5). Interestingly, the top-view AFM images of Figures 1–4 exhibit a binary phase contrast. The observed hexagonal pattern is consistent with a [111] out-of-plane orientation of the diamond lattice. However, three distinct polymer blocks are discernible in (111) level sets of the alternating diamond, as shown in Figures 6a and S4a,e.

Block copolymer films tend to bury high-surface-energy blocks below the film surface due to preferential wetting.^7^ However, the resulting thin wetting layer formed by one of the blocks can undergo solvent reconstruction,^50^ altering the composition of the surface layer in diblock copolymer^51^ and triblock terpolymer^52^ films driven by the solvent affinity of the different blocks. In the present study, PI possesses the lowest surface energy of the three blocks.^53,54^ PS and PI show similar good solubility, and PGMA shows relatively poor solubility in THF.^55^ Consequently, PS and PI are expected to segregate to the free surface of the film, burying PGMA beneath a thin surface layer of PS and PI. This arrangement contradicts the scenario shown in Figure S4e. However, removal of the PGMA phase from the (111) level set in Figure 6a produces a binary hexagonal surface pattern of PI dots within a PS matrix. This agrees with the AFM phase images, where the stiffer PS matrix appears brighter than the softer PI minority phase.^56^ At the same time, this contradicts the (111) level-set plane shown in Figure S4a, which is terminated such that the majority phase is PI.

The inherent chemical contrast in the ISG terpolymers enables DSA through the heterogeneous wettability regions on the patterned substrate.^24^ Specifically, the polar PGMA is expected to preferentially wet the silica patches on the substrate, while the nonpolar PS favors the less polar Au surface. Despite its nonpolar nature, PI also adheres to the polar silica patches, as evidenced by the cross-sectional SEM images of the PI network replicated in Au (Figure 5f). This wetting behavior is schematically shown in Figure 6a–d, which corresponds to the observed [111] out-of-plane orientation of the terpolymer diamond network and the three substrate patterns employed in the present study. Note that other (111) level sets of the alternating diamond (Figure S4a–d,e–h) are unlikely due to unfavorable wetting of the different polymer phases on the patterned substrate. Comparing Figures 2–4, it is apparent that successful DSA of ISG was achieved only for one of the three substrate patterns, namely, the patterned substrate shown in Figure 6c, and only for the smallest film thickness (120 nm). Figure 6e schematically shows the corresponding 3D alternating diamond aligned with the commensurate substrate pattern.

Comparing the registry of the (111) level set of the alternating diamond (Figure 6a) with the different substrate patterns (Figure 6b–d) allows qualitative elucidation of the DSA mechanism. The (111) level set shows a trigonal arrangement of one PI domain and three PGMA domains and vice versa. For the small patch diameters in Figure 6b, only half of the tetragonal PI–PGMA features cover the silica patches. Furthermore, only one domain is centered on a silica patch, while the three surrounding domains show minimal overlap. In contrast, when using larger silica patches, each is contacted by three domains of the tetragonal PI–PGMA array, and only one out of four PI–PGMA domains is in contact with the unfavorable Au substrate. As the silica patches are laterally diluted across the substrate, this ratio becomes increasingly unfavorable, as shown in Figure 6d. This is corroborated by the cross-sectional image in Figure 5f, where two gold struts contact each silica patch.

Because of manufacturing inaccuracies, the periodicities of the substrate patterns deviate from precise commensurability with the hexagonal pattern measured in the solvent-annealed ISG film, as shown in Figure 1b. For the silica pattern with an NND of 71 nm (Figure 6c), the adjacent polymer layer must reduce its NND by approximately 12–13%. This reduction occurs because the majority of the PI and PGMA domains wet the silica patches, which have a diameter of (50 ± 2) nm, resulting in strong pinning. Interestingly, the NND of the hexagonal polymer pattern relaxes at the film’s top surface, returning to its value observed in films on unpatterned substrates. This suggests an affine transformation of the hexagonal pattern upon transition from the substrate interface to the free interface, thus maintaining uniform order within the patterned substrate region throughout the entire film thickness, as seen in Figure 2b,e. The smaller patches shown in Figure 6b result in fewer pinned PI and PGMA domains, leading to in-plane distortions in the terpolymer surface pattern visible in Figure 3b. This behavior is similar to that of diblock copolymer spheres or lamellae in films on topographically^57,58^ or chemically^26^ patterned substrates with incommensurate periodicity. To match the pattern of Figure 6d with a silica patch NND of 134 nm, the NND of the polymer pattern needed to increase by 12%. However, the sparser pinning of PGMA and PI by the silica patches on this substrate also leads to in-plane distortions in the polymer surface pattern, as seen in Figure 3d,f.

Successful DSA of the terpolymer is observed for a film thickness of 120 nm (Figure 2) but not for the two thicker films in Figure 4. The origin of this loss in registration can be explained by the drying behavior of the polymer films. Solvent evaporation gives rise to a surface-normal solvent concentration gradient, with the lowest solvent content at the free surface.^59^ Block copolymer self-assembly, therefore, often nucleates at the free surface, propagating toward the substrate.^60^ Substrate features with strong affinities to some polymer blocks can compete with surface nucleation if the solvent concentrations at both interfaces are sufficiently close to the BCP order–disorder transition. Since the solvent concentration difference between the free and substrate surfaces increases with film thickness, substrate nucleation becomes increasingly improbable with increasing film thickness.

This is corroborated by the cross-sectional images in Figure 5. While the substrate-induced diamond morphology spans across the entire 120 nm thick film in Figure 5a, the 600 nm thick film (Figure 5d) exhibits a horizontal grain boundary, indicating the competing nucleation of the terpolymer network at both interfaces. These grains propagate in opposite directions until they meet, resulting in the formation of a grain boundary. Consequently, the pattern observed at the top surface in Figure 4e does not reflect the buried substrate patterning of the terpolymer network, as observed by the polycrystalline Moiré pattern in Figure 4e. With smaller grains, a similar polygrain structure is found for the 250 nm thick film (Figure 4d). Interestingly, the corresponding cross-sectional images (Figures 5f and S3c) lack the horizontal grain boundaries in Figure 5d. While a large set of cross-sectional images may reveal such grain boundaries, the competition of nucleation from both interfaces, combined with sufficiently fast structure propagation across the film, may result in laterally separated morphological domains spanning the entire thickness of the film, separated by vertical grain boundaries. These are not seen in the limited cross-sectional images reported in this study.

## 4 Conclusions

This study investigated the self-assembly pathway of the alternating diamond morphology (*Fd*3̅*m*, space group no. 227) in triblock ISG terpolymer films. By swelling of spin-cast polymer films in a THF solvent atmosphere followed by slow drying, a [111] out-of-plane oriented diamond network morphology was found in the ISG terpolymer films. The lateral order in these films was improved by annealing on substrates with sparse hexagonal arrays of polar silica patches on a less polar Au surface. Although the fabricated patterns deviated by about 12% from precise commensurability with the hexagonal ISG terpolymer pattern of a (111) diamond plane, uniform lateral order was induced on the entire patterned substrate of 16 × 16 μm^2^ in 120 nm thick ISG films. Cross-sectional images revealed significant lattice distortions compared to ideal cubic diamond networks, likely due to the drying protocol.

Experiments that did not result in a successful DSA provided insight into the mechanisms that govern structural alignment. When a sparse substrate pattern is used that is approximately commensurate with the polymer pattern, the substrate patches should be large enough to pin more than one of the two polymer networks. In addition, the density of the pinning patches on the substrate should not be too dilute. Furthermore, the thickness of the polymer film should be sufficiently small to suppress the competing nucleation of the terpolymer network at the free surface of the film.

To extend the DSA of self-assembled continuous network morphologies to thicker films, further optimization of the substrate pattern is required to more closely match the characteristic planes of the terpolymer network structure, and the chemical contrast of the substrate pattern should be increased. Modifying the annealing protocol may also offer control over network nucleation while reducing the observed distortions of the terpolymer network, which deviate significantly from those of ideal cubic diamond networks. These improvements promise to create templates with precisely controlled structures for fabricating advanced 3D optical metamaterials.

## 5 Experimental Section

### 5.1 Substrate Pattern Preparation

Silicon wafers were coated with a 5 nm Cr adhesive layer, followed by the deposition of a 20 nm thick Au layer. Using chemical vapor deposition, the wafers were then coated with a 10 nm SiN film to facilitate adhesion of the electron-beam resist. A 20–25 nm thick XR-1541 negative electron-beam resist was spin-coated onto the substrate, followed by an Espacer HXO_2_ anticharging coating. A JBX-9300SA electron-beam writer operating at 1 nA was used to write the patterns into the resist, followed by rinsing in deionized water for 60 s to remove the anticharging coating. Subsequently, the XR-1541 resist patterns were developed by immersion in NMD-3 for 4 min, followed by three rounds of rinsing in fresh deionized water (30 + 30 + 60 s). To allow subsequent electrodeposition, the SiN layer was etched in a NE-550 RIE system with optical endpoint detection.

### 5.2 Preparation and Solvent Vapor Annealing of ISG Films

2.5, 5, and 10 wt % solutions of the polyisoprene-*block*-polystyrene-*block*-poly(glycidyl methacrylate) (PI-*b*-PS-*b*-PGMA, ISG) terpolymer with a molar mass of 67.4 kg/mol, and volume fractions *f*_PI_ = 0.29, *f*_PS_ = 0.52, and *f*_PGMA_ = 0.19 in anhydrous anisole (Sigma-Aldrich) were spin-coated onto patterned and unpatterned substrates at 1200 rpm for 60 s with a 500 rpm/s acceleration. This resulted in approximately 120, 250, and 600 nm thick films, determined by thin film interferometry of films (see below) deposited onto unpatterned silicon substrates. The films were solvent vapor-annealed in tetrahydrofuran (THF; Sigma-Aldrich) at a maximum swelling ratio of 2.2 (i.e., the ratio of swollen to dry film thickness), followed by slow drying over 24 h, using a solvent annealing setup described previously.^38^

### 5.3 Au Replica Manufacture

The ISG terpolymer templates were voided by degrading the PI block. To this end, the samples were exposed to UV light (Fisher Scientific, λ = 254 nm, 15 W) for 15 min, followed by washing in ethanol for 30 min. The electrodeposition of Au was nucleated by cyclic voltammetry in the −0.4 to −1.15 V range at a scan rate of 0.05 V/s, followed by applying a constant potential of −0.762 V for the electrochemical growth. The remaining PS and PGMA blocks were removed by exposing the films to an O_2_ plasma (Diener electronic GmbH ZEPTO at 100 W) for 10 min.

### 5.4 Characterization

#### Film Thickness Measurements

A bifurcated optical fiber (FCR-12UV200/6002-ME) connected to an AvaLightDH-S-BAL halogen light source and a FCR-COL UV/vis collimator were used to focus light onto the sample. The reflected light was detected by an Avantes AvaSpec 2048L spectrometer that was connected to the second branch of the optical fiber, in the 500–1000 nm spectral range. Assuming a refractive index of the ISG of 1.5, the film thickness was calculated from the reflectance spectra. The swelling ratio was determined as SR = *t*/*t*_0_, where *t* is the thickness of the swollen film relative to the initial film thickness *t*_0_.

#### Atomic Force Microscopy (AFM)

AFM images of the ISG terpolymer films were acquired using a Park NX10 AFM (Park System, Suwon, Korea), which was operated in tapping mode using silicon probes (NanoAndMore) with a cantilever force constant of 40 N/m, a resonance frequency of 300 kHz, and a tip radius smaller than 10 nm. All images were scanned at a line rate of 0.7 Hz.

#### Cross-Sectional Imaging

A FEI Scios 2 (FEI, Eindhoven, The Netherlands) dual-beam field-emission scanning electron microscope (SEM) was used to obtain cross-sectional images. The FEI Scios 2 is equipped with a gallium focused ion beam (FIB), operated at 30 kV and 30 pA, that etches away the sample exposed to its beam. A Pt layer was deposited onto the film to reduce charging. SEM micrographs were acquired at a working voltage of 2 kV.

### 5.5 Diamond Nodal Surface

The literature usually provides the nodal surface approximation of the diamond with an origin choice at the (16d) Wyckoff position of space group 227 (origin choice 2 in ref (43)). This position lies on the edge of a diamond net with no vertex at the origin. It further leads to a two-term expression with no self-evident cubic symmetry; that is the *C*_3_ rotation along [111], which cyclically permutes the Cartesian components, is not immediately visible.^61^ A more symmetric choice with the same origin instead leads to a four-term parametrization.^62^ We here instead choose the origin on the (8a) Wyckoff point (origin choice 2). This yields the symmetric two-term parametrization in eq 1, in which *G* = 2π/*a* is the length of the reciprocal basis vector of the simple cubic lattice with lattice constant *a*:
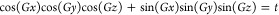


We now obtain a linear approximation for the volume fill fraction of diamond net *f*(*t*) used in the main text. A shift by 1/8·[111] centers the diamond around its midedge at Wyckoff position (16c) and yields the equation:
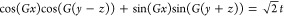
2Tailoring this expression to the second order in the Cartesian components and introducing cylindrical coordinates (ρ_c_, φ_c_, *z*_c_) with respect to the [111] edge direction describes a surface resembling a catenoid
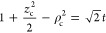
3which encloses a volume of
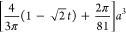
if cut at ±*a*/6 along the *z*_c_ axis. The remaining tetrahedron at the tetrahedral nodes has an edge length of . The volume correction of the network due to the nodes is therefore on the order of *a*^3^/100 and is ignored here. Since
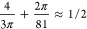
and
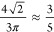
we obtain
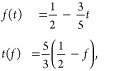
4which yields *t* = 1/3 for the measured volume fill fraction *f* = 0.3.

## Supporting Information Available

Figure S1: High-resolution AFM image of a 120 nm thick solvent-annealed ISG on a patterned substrate; Figure S2: SEM top-surface view of a Au replica of solvent-annealed ISG of varying thickness on top of patterned substrate; Figure S3: SEM images of cuts taken at an angle of γ = 90° through Au a replica of ISG films of varying thickness; Figure S4: two different calculated (111) cuts across an alternating diamond morphology and SEM images of the substrate patterns superposed with those corresponding level-set (111) cuts (PDF)

## Footnotes

1 The fill fraction of the backfilled domain in the model is increased compared to the nominal value based on the composition of the terpolymer (*f* = 0.375 instead of 0.3).
