# Supplementary material for: Directed Self-Assembly of Diamond Networks in Triblock Terpolymer Films on Patterned Substrates
Source: ACS Appl Mater Interfaces. 2023 Nov 21;15(50):57981–91. doi: 10.1021/acsami.3c10619 (PMC10739600; doi:10.1021/acsami.3c10619)
Supplement: Supplementary file 1 — am3c10619_si_001.pdf [file am3c10619_si_001.pdf]

Directed self-assembly of diamond networks in triblock terpolymer films on patterned substrates – Supporting Information –

Doha Abdelrahman,[†] René Iseli,[†] Michimasa Musya,[‡] Butsurin Jinnai,[¶] Shunsuke Fukami,[‡,§,¶,‖,⊥] Takeshi Yuasa,[#,△] Hiroaki Sai,[#,▽] Ulrich B. Wiesner,[#] Matthias Saba,[†] Bodo D. Wilts,[†,@] Ullrich Steiner,[†] Justin Llandro,[*,‡,§] and Ilja Gunkel[*,†]

†*Adolphe Merkle Institute, University of Fribourg, Chemin des Verdiers 4, 1700 Fribourg, Switzerland*

‡*Laboratory for Nanoelectronics and Spintronics, Research Institute of Electrical Communication, Tohoku University, 2-1-1 Katahira, Aoba-ku, Sendai 980-8577, Japan*

¶*WPI Advanced Institute for Materials Research, Tohoku University, 2-1-1 Katahira, Aoba-ku, Sendai 980-8577, Japan*

§*Center for Science and Innovation in Spintronics, Tohoku University, 2-1-1 Katahira, Aoba-ku, Sendai 980-8577, Japan*

‖*Center for Innovative Integrated Electronic Systems, Tohoku University, 468-1 Aramaki Aza Aoba, Aoba-ku, Sendai 980-0845 Japan*

⊥*Inamori Research Institute for Science, Kyoto 600-8411, Japan*

#*Department of Materials Science and Engineering, Cornell University, 214 Bard Hall, Ithaca, NY 14853-1501, USA*

@*Department of Chemistry and Physics of Materials, University of Salzburg, Jakob-Haringer-Str. 2a, 5020 Salzburg, Austria*

△*Current affiliation: Yokkaichi Research Center, JSR Corporation, Yokkaichi, Mie 510-8552, Japan*

▽*Current affiliation: Simpson Querrey Institute for Bionanotechnology, Northwestern University Evanston, IL 60208, USA*

E-mail: llandro.justin.b6@alumni.tohoku.ac.jp; ilja.gunkel@unifr.ch

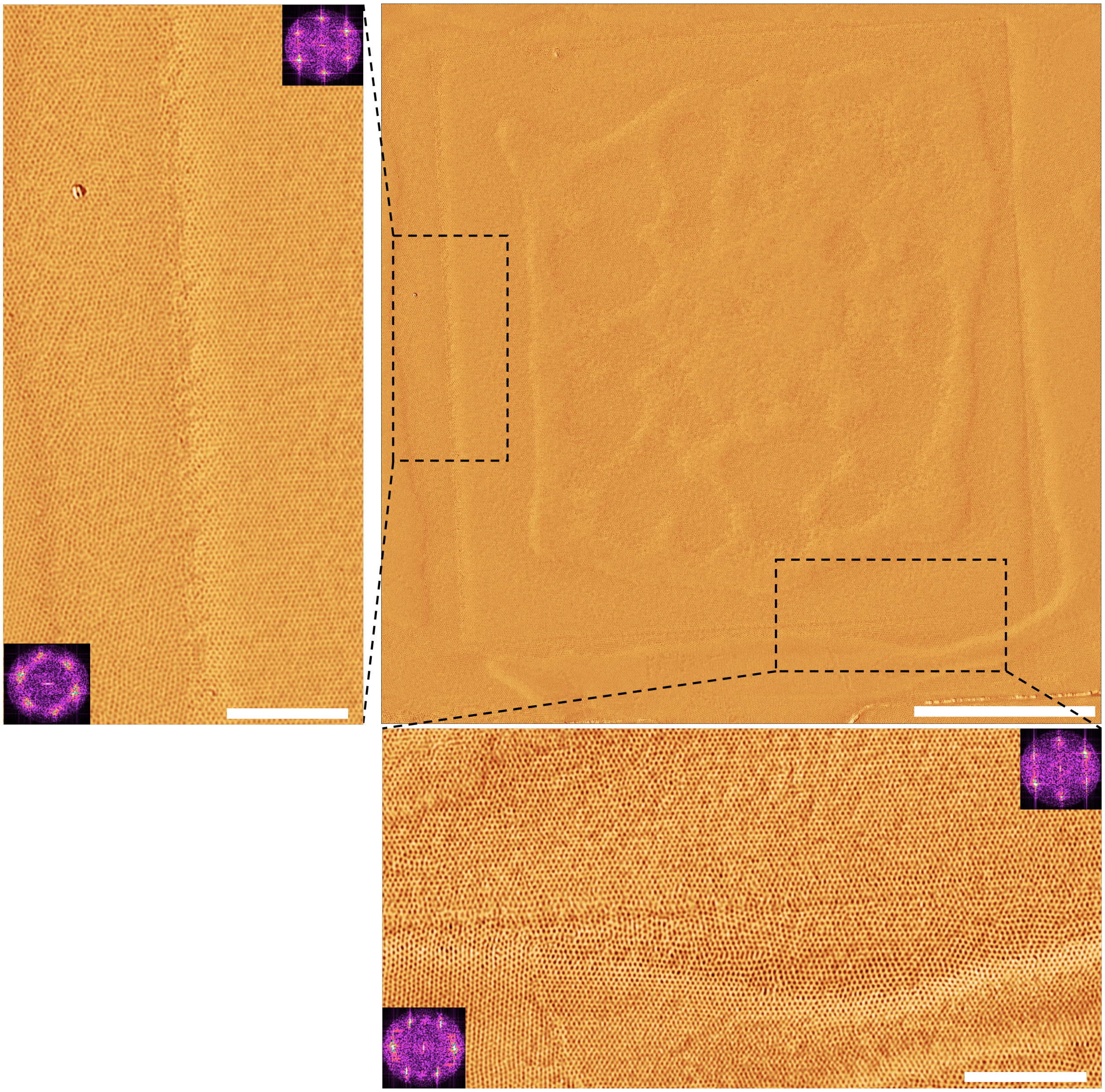

Figure S1: High-resolution AFM image of a 120 nm thick solvent annealed ISG on a patterned substrate. The dashed rectangles highlight magnified regions of the terpolymer surface pattern across the border of the patterned substrate. The square shape of the substrate pattern is clearly discernible in the main image. Insets show corresponding FFTs, confirming uniform order throughout the film on the patterned substrate, with the orientation matching that of the underlying substrate pattern at different regions. Conversely, the terpolymer pattern on the unpatterned substrate region shows grains with various orientations. The film occasionally exhibits terracing in its thickness, as evident in the lower region of the bottom image. Scale bars: 5 µm (square image), 1 µm (magnified views).

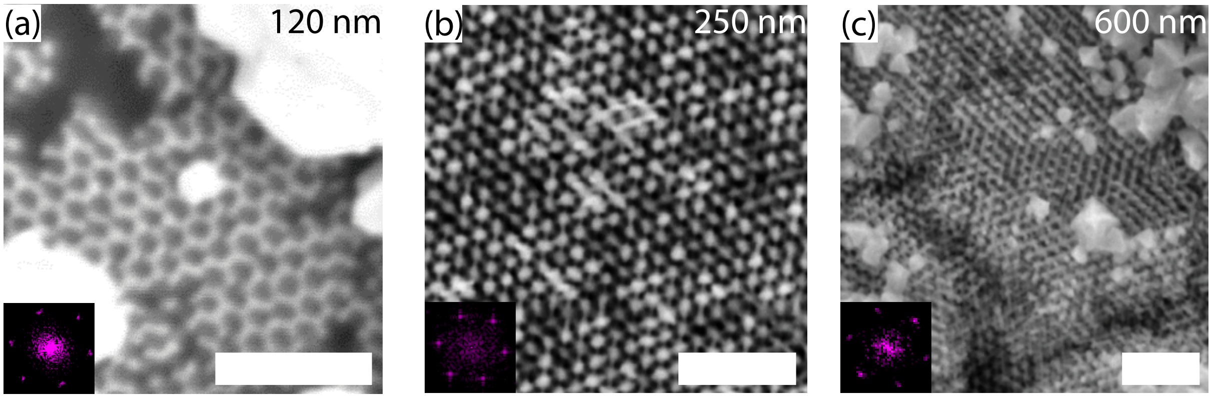

Figure S2: SEM top-surface view of Au replica of (a) 120 nm, (b) 250 nm, and (c) 600 nm thick solvent annealed ISG on top of patterned substrate. The insets show the corresponding FFTs. Scale bars: 250 nm.

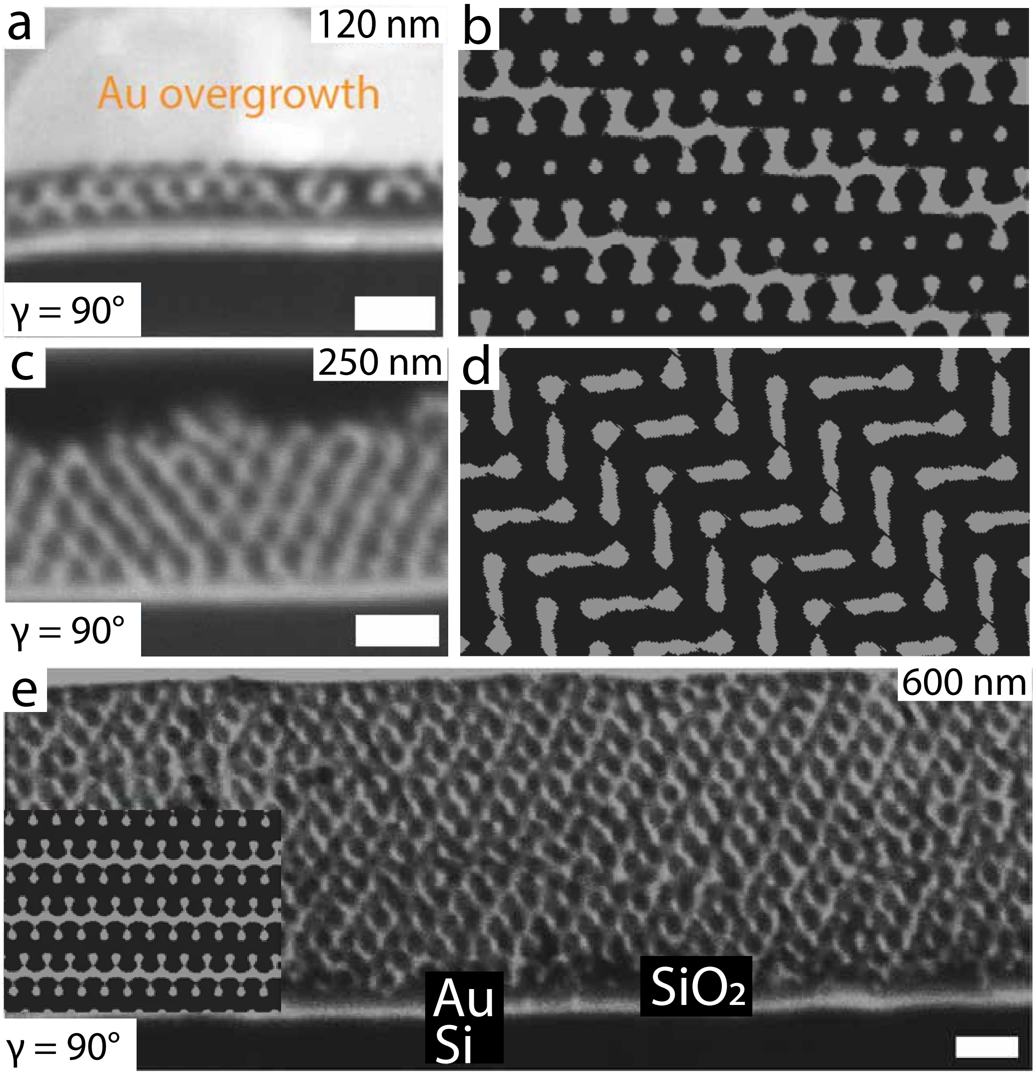

Figure S3: SEM images of cuts taken at an angle of $\gamma = 90°$ through Au replica of (a) 120 nm, (c) 250 nm, and (e) 600 nm thick ISG films. The angles of the diamond level-sets (fill fraction $f = 0.375$) with respect to the (111) plane are (b) 87° for the $(54\bar{8})$, (d) 64° for the $(9\bar{2}0)$, and (e) 90° for the $(11\bar{2})$ plane. Scale bars: 100 nm.

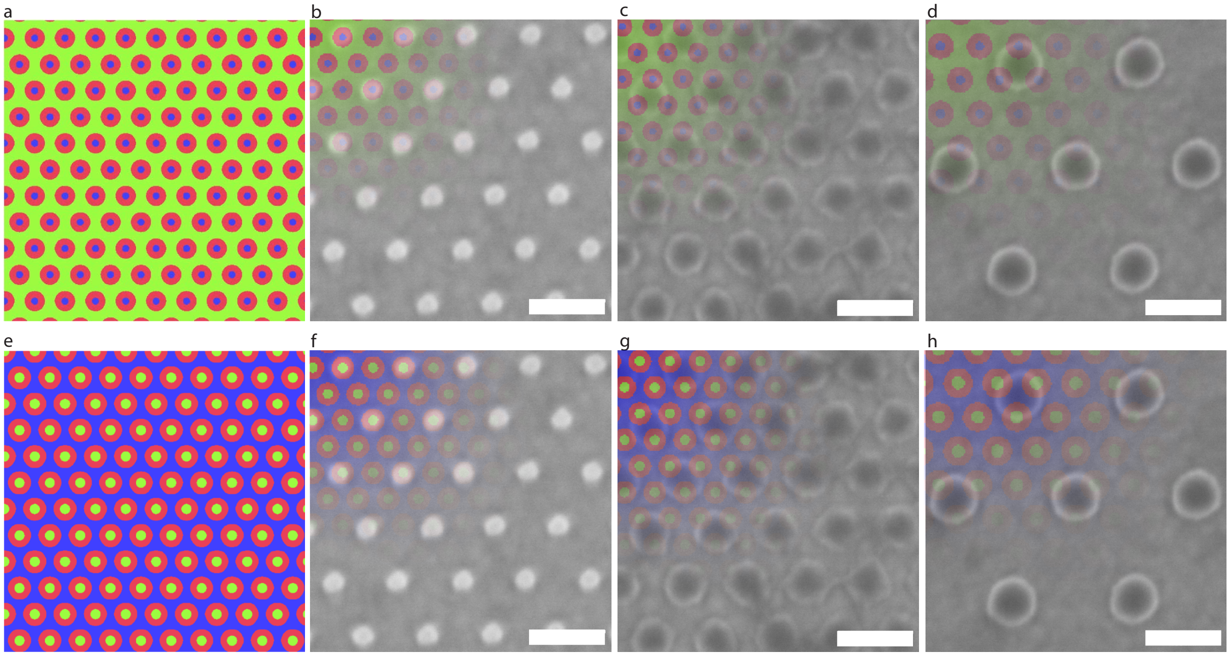

Figure S4: (a,e) two different calculated (111) cuts across an alternating diamond morphology (red = PS, green = PI, and blue = PGMA). (b–d, f–h) SEM images of the substrate patterns superposed with the corresponding level-set (111) cuts, shown in (a,e), respectively. The dimensions of the substrate patterns are (b,f) 71 ± 3 nm NND, 25 ± 2 nm patch diameter, (c,g) 71 ± 3 nm NND, 50 ± 2 nm patch diameter, and (d,h) 134 ± 3 nm NND, 60 ± 2 nm patch diameter. The superposed level-set images are compressed by 13% in (b,c,f,g,j,k) and stretched by 12% in (d,h). Scale bars: 100 nm.
